# The interplay between job demands & resources and mental health: a novel approach using hidden Markov models

**DOI:** 10.1007/s10865-025-00626-2

**Published:** 2026-01-19

**Authors:** Stef Bouwhuis, Ceciel Pauls, Mauricio Garnier-Villarreal, Dimitris Pavlopoulos

**Affiliations:** 1 Department of Sociology, Vrije Universiteit Amsterdam, De Boelelaan 1105, 1081 HV Amsterdam, The Netherlands; 2 Department of Health Sciences, Vrije Universiteit Amsterdam, Amsterdam, The Netherlands

**Keywords:** Hidden Markov models, Job demands and resources, Mental health, Healthy worker effect

## Abstract

We use a novel method (cross-lagged hidden Markov models) to identify which combinations of job demands and resources occur among workers, how often, and how these affect mental health and vice versa. Hidden Markov models (HMM) are a longitudinal extension of latent class analysis (LCA), which can be used to measure concepts that are not directly observable. As in LCA, indicator variables are used to measure such concepts. We use twelve indicators of JDR, and five indicators of mental health. HMMs group individuals with similar response patterns on the indicators in categories of the latent variable and analyse how individuals move between these categories. Additionally, predictors can be added to the model to investigate which factors influence transitions between the identified states. We used this model to study the cross-lagged relations between JDR and mental health: how JDR in time point $t-1$ affects mental health in time point *t* and mental health in time point $t-1$ affects JDR in time point *t*. We used yearly data from the Dutch Longitudinal Internet Social Survey (LISS) from 2016 to 2023. Our sample includes respondents who were employees in 2016 and for whom we had data on their JDR and mental health for at least four years. We identified six JDR states, ranging from ‘Tough job’ (high demands and few resources) to ‘Dream job’ (moderate demands and very high resources). We also identified three mental health states: poor, moderate, and good. Among those in moderate health, transitions to good health were more common for respondents in the ‘Dream job’ state and less common for respondents in the ‘Tough job’ state. Our results suggest a healthy worker effect: transitions from states with a disadvantageous combination of JDR to better states were more common among employees in moderate or good mental health. Our study shows how HMMs can improve our knowledge on the empirical predictions of widely studied theories such as the JDR model and its interplay with mental health. This is relevant for scholars and practitioners alike.

## Introduction

In many countries, a substantial part of the working population experiences (work-related) mental health problems. In the Netherlands, 31.5% of the workforce experience work-related mental health problems - i.e. health problems that originated at work or were aggravated at work^1^ - while 14% experience symptoms of burnout.^2^ Although the issue is particularly pressing in the Netherlands, this is also the case in other EU countries: on average, 15% of the EU workforce faces mental health problems.

The Job Demands and Resources (JDR) model is a widely used theoretical framework for studying the relationship between work characteristics and mental health. According to this framework, two psychological processes relate work characteristics to mental health (Bakker & Demerouti, 2007). The first process is the burnout process. In this process, job demands, such as work pressure and emotional and mental demands, result in stress or strain, which in turn affects the mental health of workers. A high level of these job demands depletes workers and may ultimately contribute to the development of burnout complaints or other mental health problems. The second process is the motivational process. In this process, job resources, such as autonomy and support from colleagues and supervisors, result in higher job motivation, which, in turn, can also be related to mental health. The availability of these resources increases employees’ motivation and dedication, and results in better mental health. These two processes also influence one another. High job demands may be mitigated by high job resources. In other words, high job demands may not result in declining mental health if they are accompanied by high job resources. In addition, high resources may not result in high levels of motivation if job demands are high as well.

Empirical research on the relationship between job demands and resources and the well-being of employees provides ample support for the theoretical predictions of the JDR model (Lesener et al., 2019; Bakker et al., 2023). Most previous research on JDR has been variable-centred, meaning that it has studied the relation between variables. Recently, studies have employed person-centred approaches to investigate the relationship between JDR and health. Portoghese et al. (2025), for instance, employed Latent Class Analysis to study the combinations of job demands and resources that South Korean nurses experience. They identified five classes of nurses. The classes primarily differed in terms of job demands; resources were relatively similar across the classes. They also found that these classes were related to work-related well-being: nurses in (severely) demanding jobs more often reported physical and emotional exhaustion. Van den Broeck et al. (2012) also use a cluster technique to identify groups of employees that experience similar demands and resources among Flemish employees. They identified four profiles and, like Portoghese et al. (2025), found that demanding jobs (with high demands and low resources) are related to the lowest employee well-being. Lastly, Mäkikangas et al. (2021) identify groups of Finnish white-collar employees based on their burnout symptoms and subsequently studied the relation with job demands and resources.

As the three studies above argue, it is important to examine which combinations of demands and resources employees actually experience and how these are related to health and well-being. This can help design interventions that target combinations of demands and resources that occur in real life. In addition, such studies can contribute to the JDR model theoretically, as they can shed new light on how interactions between different jobs and resources affect health and well-being. However, previous person-centred studies on the relation between JDR and health are predominantly cross-sectional. As a result, it is difficult to determine the direction of the relation between job demands and resources and mental health. Theoretically, the JDR model is one-directional in its predictions regarding the relation between work and health, i.e. it attempts to explain how job demands and resources influence (mental) health of workers, but not vice versa. However, the relationship between work characteristics and health may be bidirectional. Job demands and resources affect mental health, but a healthy worker effect may exist as well (Li and Sung, 1999). The healthy worker effect is a form of selection and describes that, in comparison to the general population, the workforce is generally healthier (Chowdhury et al., 2017). The healthy worker effect consists of two components: the healthy hire effect and the healthy survivor effect. The healthy hire effect describes that healthy workers are more likely to be hired in certain scenarios, or that unhealthy individuals may avoid certain workplaces and occupations (Nordström et al., 2016). The *healthy-survivor effect* describes that unhealthier employees are more likely to leave certain workspaces and occupations (Nordström et al., 2016). Thus, while JDR might affect mental health, the opposite might also be true. Thus, longitudinal evidence is key to understanding both the potential short- and long-term impact of health selection on employment status (Wagenaar et al., 2012).

The aim of this study is to address the shortcomings of previous research on the JDR model by using a novel approach to study the relationship between job demands and resources and mental health. Specifically, we address two research questions. First, what combinations of demands and resources exist among workers in the Netherlands, and how often do they occur? Second, how are these combinations of job demands and resources related to mental health? To address these questions, we applied a cross-lagged hidden Markov model (HMM). Hidden Markov models belong to the family of latent variable models and are, in fact, a longitudinal extension of Latent Class Analysis (LCA). LCA can be used to measure categorical latent variables, i.e. concepts that are not directly observable. Indicator variables are used as imperfect measures of these concepts. LCA is a person-centred approach, which means that individuals are probabilistically^3^ grouped, based on their scores on these indicator variables. This results in a categorical latent variable, where each category represents a group of individuals with similar values in the indicator variables. LCA is particularly suitable for measuring multidimensional concepts such as JDR. In an HMM, observations are first clustered in categories (which are referred to as ‘states’ in HMM) based on their scores on the indicator variables. In our case, we identify states of individuals with similar job demands and resources, and states of individuals with similar mental health. Subsequently, we model how individuals transition between these states and how these transitions are influenced by mental health, and vice versa. In more detail, we introduce cross-lagged effects in HMM, which means that we model how mental health in *t*-1 is related to transitions in JDR from *t*-1 to t and how JDR in *t*-1 is related to transitions in mental health from *t*-1 to *t*.

## Methods

### Data and study population

We utilised the Longitudinal Internet Social Survey (LISS) to investigate the relationship between job demands and resources, and mental health. LISS is an online panel consisting of around 7,500 individuals in the Netherlands, clustered in 5,000 households (Scherpenzeel & Das, 2011). They are asked to participate in annual questionnaires covering various topics on both the individual- and household-level, including demographic information, work and schooling, and health. In addition, they are asked to provide background information on a monthly basis. For the present study, we linked the work and schooling module to the health and background modules. We study the relationship between JDR and mental health among so-called prime-age workers, that is, workers between the ages of 25 and 55. This decision was made to exclude workers still in education and workers approaching or reaching the statutory retirement age during the study period. We chose 2016 as the baseline measurement and included individuals in paid employment or self-employment in that year. We followed these individuals for seven years, that is, until 2023. We selected individuals who participated in at least three follow-up measurements and had at least four years of health data. Of the 2163 individuals in paid employment in 2016, 1140 (52.7%) matched these criteria. Individuals excluded because they did not meet our criteria regarding the minimum number of waves were more likely to be women (55%), younger (with an average age of 40 years compared to 42 years for those included in the sample), and more often attended only primary or secondary school. The 1140 individuals in our study came from 946 different housholds. This means that some individuals in our population were part of the same household. In our study, 752 households had one member in our study population (79% of all households) and 194 housholds had two members in our study population (21% of all households). This means that of the 1140 people in our study population, 388 individuals reside in a household of which two members were included in our study. The remaining individuals (*n* = 752) come from a household of which only one member was included.

### Job demands and resources

We used four indicators of job demands, namely physical, mental, and emotional demands as well as work pressure (see online appendix, https://osf.io/ydxz4). In addition, we included six indicators of job resources: the ability to work at one’s own speed, autonomy, opportunity to learn, support, appreciation, and wages. Some of these indicators were measured as frequency (never, sometimes, often), and other indicators were presented to respondents in the form of statements. Respondents were asked to indicate the extent to which they agreed with these statements (disagree entirely, disagree, agree, agree entirely).

In addition, we included two indicators that could either be a demand or a resource. First, contract type, which had the following categories: no contract, permanent, fixed-term, and agency. A permanent contract was considered a resource since it provides job security and can be interpreted as a sign of appreciation. Other types of contracts were considered to be a demand. Second, the frequency of working from home (less than one day a week, around one day a week, more than one day a week, no). Previous research has indicated that working from home could be a resource, especially when it substitutes working on site (Yang et al., 2023). On the other hand, it can result in difficulties combining work responsibilities and domestic work, and continuous demand on digital up-skilling can be considered a demand (Collins et al., 2016).

### Mental health

We assessed mental health using the five indicators from the Mental Health Inventory 5 (MHI5) scale. The MHI5 assesses an individual’s mood over the past month. Individuals were asked how often they felt a) very anxious, b) so down that nothing could cheer them up, c) calm and peaceful, d) depressed and gloomy, and e) happy. Participants were presented with the following answer categories: never, seldom, sometimes, often, mostly and continuously. Furthermore, we included an indicator regarding the use of medication for anxiety or depression over the past two weeks (‘*I am currently taking medication at least once a week for anxiety and depression*’). In the past, this variable has been used to create latent states of mental health (van der Velden et al., 2019, 2022)

### Analytical strategy

In this paper, we apply a cross-lagged hidden Markov model to estimate the relationships over time between two categorical latent variables: one representing mental health (MH) and another one representing job demands and resources (JDR). This means that we treat job demands and job resources as one latent construct, with two dimensions (job demands and job resources). Theoretically, it could be argued that job demands, on the one hand, and job resources, on the other, are two separate theoretical concepts that set in motion two distinct theoretical processes. Based on this, treating them as two latent variables might be preferable. However, treating job demands and resources as a single latent variable had the benefit of being able to distinguish meaningful subpopulations with similar patterns of job demands *and* resources resembling real-world work settings in which employees experience both demands and resources at the same time. Our approach is in line with other studies that use a person-centred approach to study job demands and resources (Portoghese et al., 2025; Van den Broeck et al., 2012)

HMM is a mixture model with a dynamic categorical latent variable ($x_{it}$). Hidden Markov Models (HMM) are also called Latent Transition Analysis (LTA) or Latent Markov Model (LMM) (Nylund-Gibson et al., 2023; Vermunt, 2010). The first characteristic of this model is that the state transitions over time are modelled with a first-order Markov structure. This means that the realisation of the latent state at time point *t* is only determined by its realisation at time point $t-1$. The second characteristic is that the latent states are imperfectly measured by multiple observed indicators. In this way, the categories of the latent variable represent unobserved groups that present the highest levels of within-group homogeneity and between-group heterogeneity (Biemer, 2011; Vermunt, 2010).

The HMM model consists of two main parts, the measurement and the structural part. The measurement part ($P({{y}_{itj}|{x}_{it}}=k_{t})$) represents the item response probabilities for each item *j* across time points *t*, for estimated latent classes *k*. The item response probabilities are conditional on the categorical latent variable ${x}_{it}$ with a fixed number of latent states *k*. The structural part of the model represents the pattern of latent-state dynamics over time. These dynamics are described by the initial state probabilities ($P({{x}_{i0}}=k_{0})$), and the transition probabilities from time $t-1$ to time *t* ($P({{x}_{it}}=k_{t}|{x}_{it-1}=k_{t-1})$).

The basic HMM assumes that the transition probabilities are time-invariant, so e.g. between time 1 and time 2, we have the same probabilities as between time 5 and time 6. We relax this (possibly unrealistic) assumption by including *year*/*time* as a predictor of transition probabilities ($\boldsymbol{Z_i}$). In this way, we estimate time-varying transition probabilities.

We use HMM as an exploratory method, which first requires us to define the best measurement model for each of the two factors (MH and JDR). We achieve this by estimating a sequential set of HMMs with an increasing number of latent states *k* - ranging from 1 to 10. Then, we evaluate the relative model fit with information criteria, i.e. Akaike Information Criteria (AIC) and Bayesian Information Criteria (BIC). Based on these indices, we select two or three possible solutions with the most likely number of latent states and then evaluate the theoretical interpretability of each solution. Thus, the class enumeration process involves an evaluation of a combination of statistical and theoretical information (Masyn, 2013; Van Lissa et al., 2024). In addition, we took into account the sizes of states. Considering the sample size, we preferred solutions in which all states contained more than 10% of the study population to ensure a sufficient number of respondents in each of the states.

After we chose a number of states for each factor, we tested the assumption of longitudinal measurement invariance. We extended the steps from multiple-group measurement invariance in LCA (Kankaraš et al., 2018), comparing the BIC from a model with time as a predictor in the structural part of the model, with an alternative model with time also as a predictor of each indicator in the measurement part of the model. For both factors, we found that the model in which time as added as a predictor of the indicators had a larger BIC value, which indicates worse model fit. Therefore, we chose to assume longitudinal measurement invariance.

Once we select the most likely number of latent states for each factor, i.e., JDR and mental health, we proceed with estimating the cross-lagged HMM. For this phase, we adopt a two-step approach for the estimation of the relations in the structural part of the model. In the first step, we fix the parameters of the measurement part of the model so that they do not change due to the addition of cross-lag effects (Bakk & Kuha, 2021; Lyrvall et al., 2024). In the second step, we add the state of one factor at $t-1$ as a predictor of the other factor at time *t*. In other words, the state at time *t* is directly predicted by the state of the same factor at time $t-1$, and by the state of the other factor at time $t-1$.

 Because 388 individuals in our study came from households with two members in our study population, we have a multilevel structure of subjects nested within households. In order to evaluate the effect of this nested structure, we estimated the HMM models with a random intercept on the measurement model for each indicator. As the AIC and BIC improved, we decided to estimate all models with the random intercept that accounts for the variability related to the multilevel structure (Hox et al., 2018).

The final model includes time-varying transition probabilities for MH conditional on each lagged JDR state, and for JDR conditional on each state of lagged MH. Emotional demands was removed as an indicator of JDR from the final model for several reasons. First, with every model, it presented very low factor loadings (below 0.1), indicating that it does not help to distinguish between classes. Second, it showed a high correlation with the indicator mental demands, as they were providing redundant information. So we decided to keep the initiator of mental demands, and remove the one for emotional ones. All analyzes were performed with LatentGOLD, version 6.0 (Vermunt & Magidson, 2016).

At the first time point the item’s missing data ranged between 2% and 5%, while at the last time point missing data ranged between 18% and 23%. Presenting a pattern of attrition common in longitudinal research. To handle missing data under the assumption of missing at random (MAR) we used Full Information Maximum Likelihood (FIML) estimation (Enders, 2022).

## Results

### Description study population

Most people^4^ in our sample had a permanent contract (around 82%) (see Appendix). Of the job demands, mental demands were most commonly experienced (95% reported experiencing mental demands often or sometimes, compared to 37% for physical demands and 35% agreed with experiencing work pressure). Concerning most resources, the majority of respondents reported that they experienced them. Autonomy in work was experienced most frequently: more than 80% of the respondents stated having autonomy. In addition, almost all respondents reported that they can work at their own speed at least sometimes and 63.3% of are able to do so often. In addition, 77.6% of the respondents reported receiving support in difficult situations and 77.9% experienced learning opportunities, 66.2% mentioned receiving sufficient wages, and 70.2% experienced appreciation at work.

### Describing job demands and resources states

**Fig. 1:**
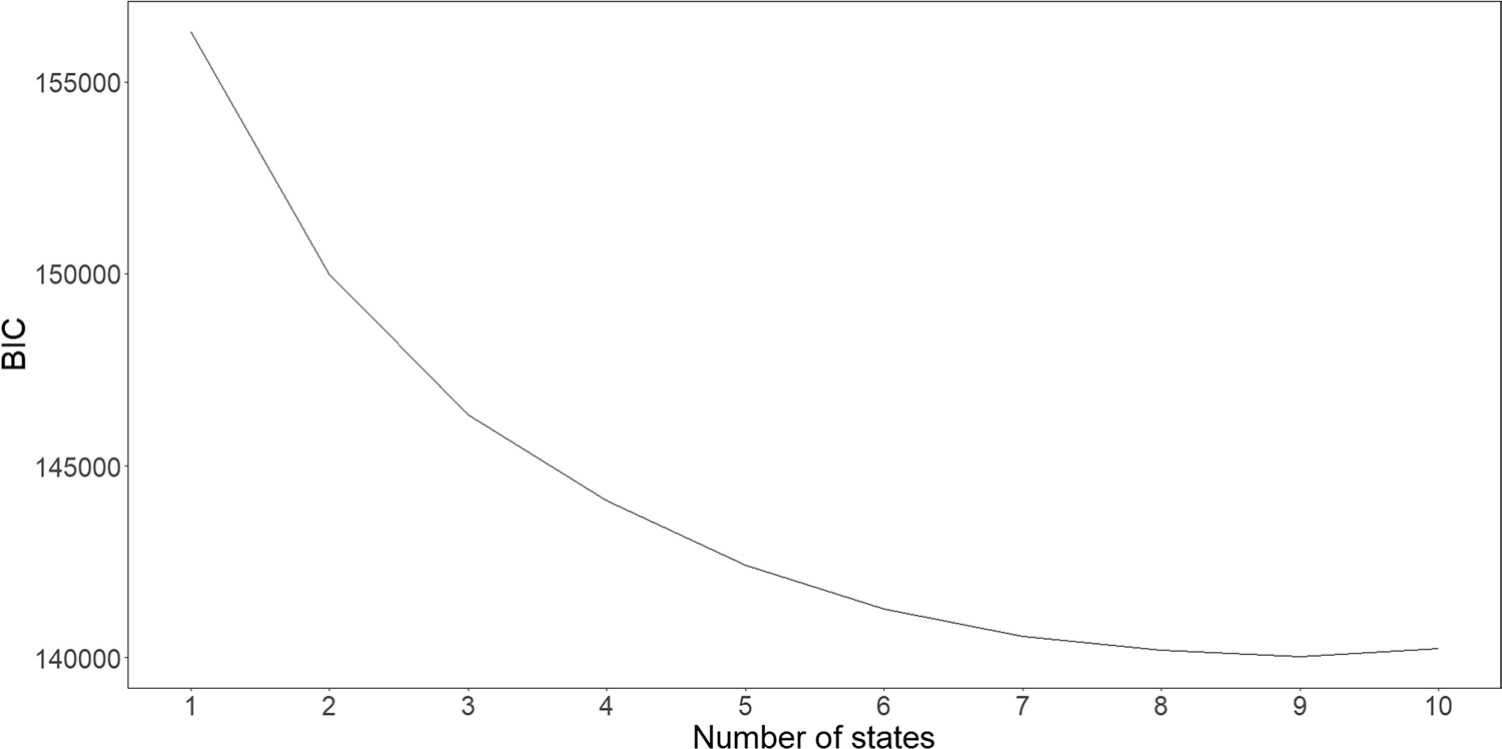
BIC for models with increasing number of JDR states

Figure 1 shows the Bayesian Information Criterion (BIC)^5^ for models with a different number of states for job demands and resources. It shows that the model with two states has a considerably lower BIC and AIC than the model with one state, indicating a better model fit. From three states onward, the decrease in BIC and AIC is smaller. Thus, we examined the theoretical interpretability of the models with two to seven states. Since the seven-state model included two states that contained less than 10% of the sample, with the smallest state consisting of approximately 6.6%, we decided against this model. We selected the six state model as the final model. Compared to models with fewer states, this model had two advantages. First, the six-state solution included a state comprised of respondents with very high levels of resources that was not identified in models with fewer states. Second, the states were more clearly separated on the working-from-home indicator than in models with fewer states.

**Fig. 2:**
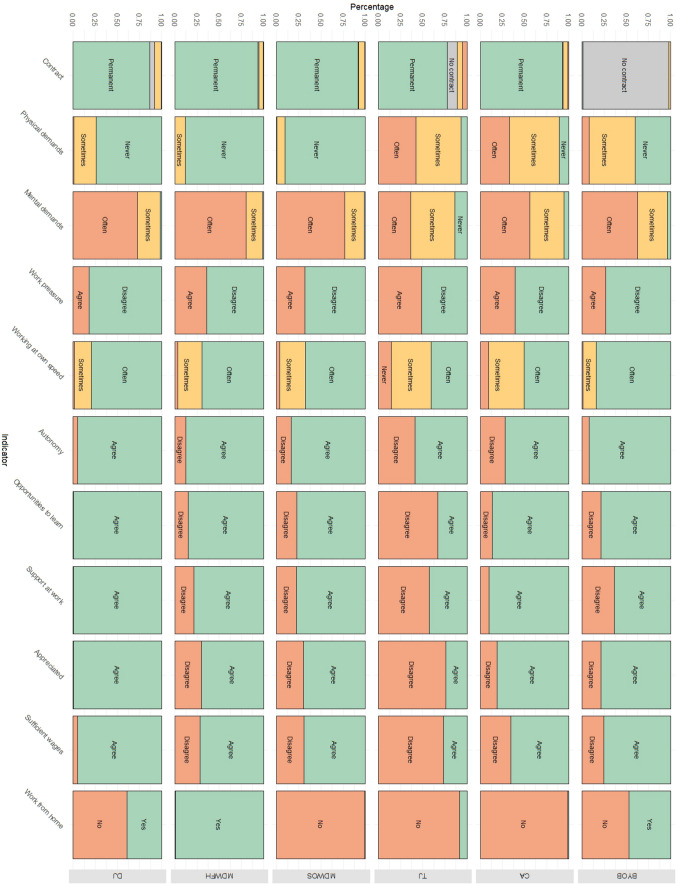
Six JDR states

In Fig. 2 shows the six states of job demands and resources we identified. For each state, it is shown how individuals in this state are distributed over the different categories of the indicators. To increase the readability of the figure, the categories ‘Completely agree’ and ‘Agree’ were merged as well as the categories ‘Completely disagree’ and ‘Disagree’. In addition, we merged the categories of the working from home indicator. The detailed results are included in the online Appendix (https://osf.io/ydxz4).

Each row in Fig. 2 represents one of the six states of JDR we identified. For each indicator, it is shown how the respondents in the six states are distributed over the categories. For instance, the top row is the ‘BYOB’ state. As is visible on the far left bar in the figure, almost all individuals in this state do not have an employment contract. The bar on the right next to it shows that about a third of the respondents in this state report never experiencing physical demands.

Below, the six states will be discussed in more detail.

In three of the six identified states a relatively high proportion of respondents reported experiencing physical work demands often or sometimes. These states are shown in the top three rows of Fig. 2. The first state that was characterized by relatively high levels of physical demands contained around 10% of our sample. We labeled this state ‘Being your own boss’ (abbreviated as BYOB), since it stands out because almost 97% of the respondents do not have an employment contract. Descriptive analyses showed that around 68% of the individuals in this state was self-employed. Approximately 8% of the respondents in this state reported experiencing physical demands often, and 52% reported experiencing these demands sometimes. In addition, this state is characterized by the highest share of respondents stating that they often can work at their own speed (84%) and the high share of people experiencing autonomy (92%).

The second state - in which around 26% of our respondents were grouped - was labeled ‘Challenged & appreciated’ (abbreviated as CA). In this state, the proportion of respondents who report that they often experience physical demands is also high (around 33%). However, compared to the previous state, fewer respondents experienced work pressure, while more respondents had access to work resources. For example, 90% reported experiencing support at work, 81% feeling appreciated, and around 65% believed that they receive sufficient wages.

The third one of these states, comprising around 16% of our sample, is characterized by high physical demands and work pressure, as well as a low level of resources. For this reason, we label this state ‘Tough job’ (abbreviated as TJ). In this state, 43% of individuals reported that they often experienced physical work demands, while many also experienced work pressure. In contrast, a relatively small share of respondents experienced the availability of job resources. Specifically, only around 33% reported opportunities to learn, 24% mentioned feeling appreciated, and around 27% believed they received sufficient wages, considering their effort and performance.

The remaining three states are characterized by relatively low physical demands but relatively high mental demands compared to the states discussed above. We labeled the first of these states, the fourth row in Fig. 2), ‘Mentally demanding work from home’ (abbreviated as MDWFH). Around 20% of the respondents are grouped in this state. In this state, a relatively high share of respondents (around 36%) reported work pressure. This state also stood out because roughly 99% of the respondents reported working from home at least one day per week.

The second state with low levels of physical demands, containing around 21% of our sample, is similar to the previous, but individuals never work at home. For this reason, we labeled this state ‘Mentally demanding work on site’ (abbreviated as MDWOS). The share of respondents experiencing work pressure is relatively high but slightly lower than in the Though job state (around 32%).

The third of these states and the last row in Fig. 2 includes individuals (around 8% of our sample) who experience low demands (with the exception of mental demands that are close to the average) and a high availability of resources. The latter is the most distinguishing feature of this state. For example, 100% of the respondents report that they feel appreciated and that they receive support. Around 95% reports receiving sufficient wages. Therefore, we labeled this state ‘Dream job’ (abbreviated as DJ).

**Fig. 3:**
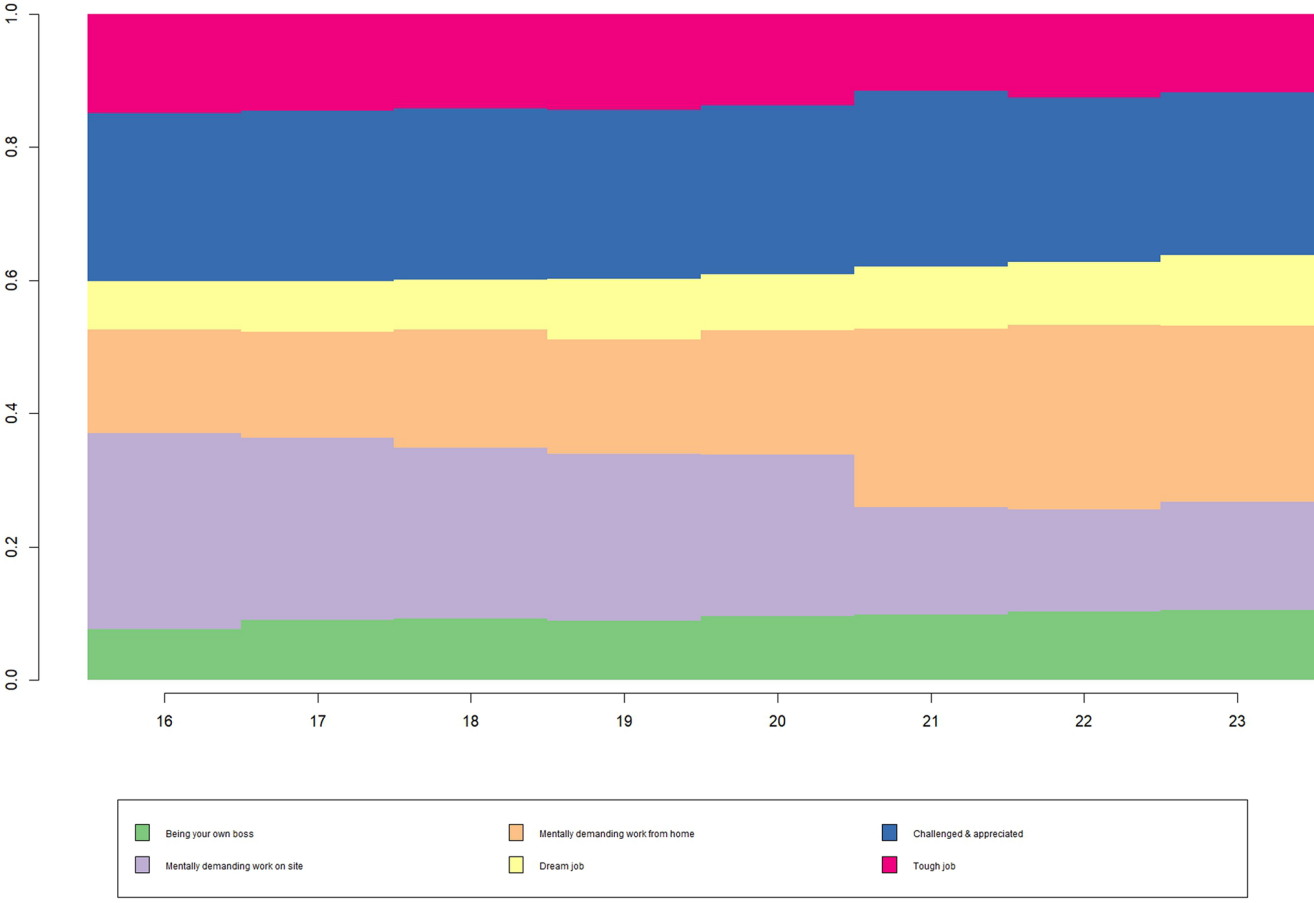
Density plot for JDR states: the share of respondents in each of the states in each year

The share of respondents in each of the six JDR states discussed above refers to the pooled share across all eight time points. Figure 3 shows how this share evolves over time. To the left, the share of workers in each of the six states is equal to the initial probabilities, i.e. the share of workers in each state in 2016. The figure shows that the share of workers in the ‘Tough job’ state and the ’Mentally demanding work on site’ declines (the latter particularly in 2020, which is most likely caused by the Covid-19 pandemic). The share of respondents in the ‘Mentally demanding work from home’ and ‘Being your own boss’ states increases over the years.

Figure 3 presents the distribution of respondents in the six states at the different time points, but not how each respondent moves between these states. However, HMMs do provide insight into these transitions. There are different ways to present these. In this paper, we first discuss transition probabilities, which quantify the degree of mobility between JDR states, and then show index plots. Table 1 shows the average transition probabilities for the JDR states over the study period. It is important to note that the model estimates yearly transition probabilities, representing the likelihood of moving from one state to another between one year and the next. The transition probabilities presented in Table 1 are the average probabilities over the study period.

**Table 1:** Average yearly transition probabilities between JDR states over the study period

	JDR-state *t*					
JDR state $t-1$	BYOB	CA	TJ	MDWFH	MDWOS	DJ
BYOB	96.4%	0.4%	0.7%	0.9%	0.7%	0.9%
CA	0.5%	93.0%	4.1%	0.5%	0.7%	1.3%
TJ	0.8%	7.9%	87.6%	1.0%	1.5%	1.2%
MDWFH	1.3%	0.2%	0.3%	90.0%	3.8%	4.3%
MDWOS	0.6%	0.7%	0.4%	10.7%	84.3%	3.4%
DJ	1.1%	2.6%	1.5%	8.0%	8.3%	78.7%

**Fig. 4:**
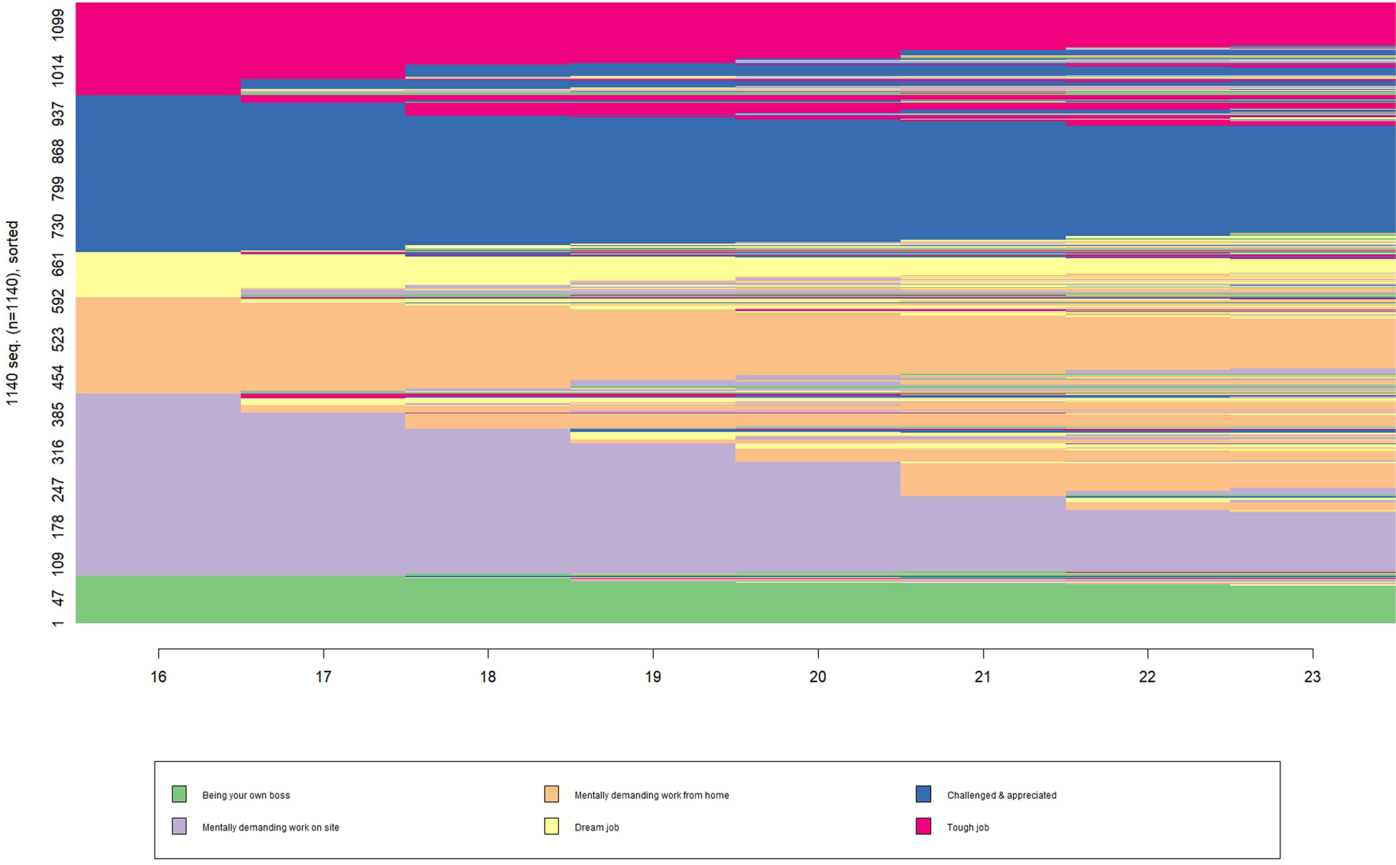
Index plot for the JDR states: each horizontal line represents the transitions of one respondent

Figure 4 shows the index plot for the modal predicted JDR state. Each horizontal line represents one of the respondents (N=1,140) in our study. This figure illustrates that those in the ‘Being your own boss’ state are likely to remain in this state, which aligns with the high staying probabilities (96.4%) for this state in Table 1. Workers in the ‘Challenged & Appreciated’ state are also likely to remain in the same state (93.0%), as are workers in the ‘Mentally demanding work from home’ state (90.0%). On the other hand, workers who start in the ‘Mentally demanding work on site’, and ‘Dream job’ state are less likely to stay in the same state from one year to the next (84.3%, 78.7% respectively). Individuals classified in the first of these groups are likely to transition to the ‘Mentally demanding work from home’ state. Those in the ‘Dream job’ state are likely to move to either of the mentally demanding states, but also to other states.

### Describing mental health states

**Fig. 5:**
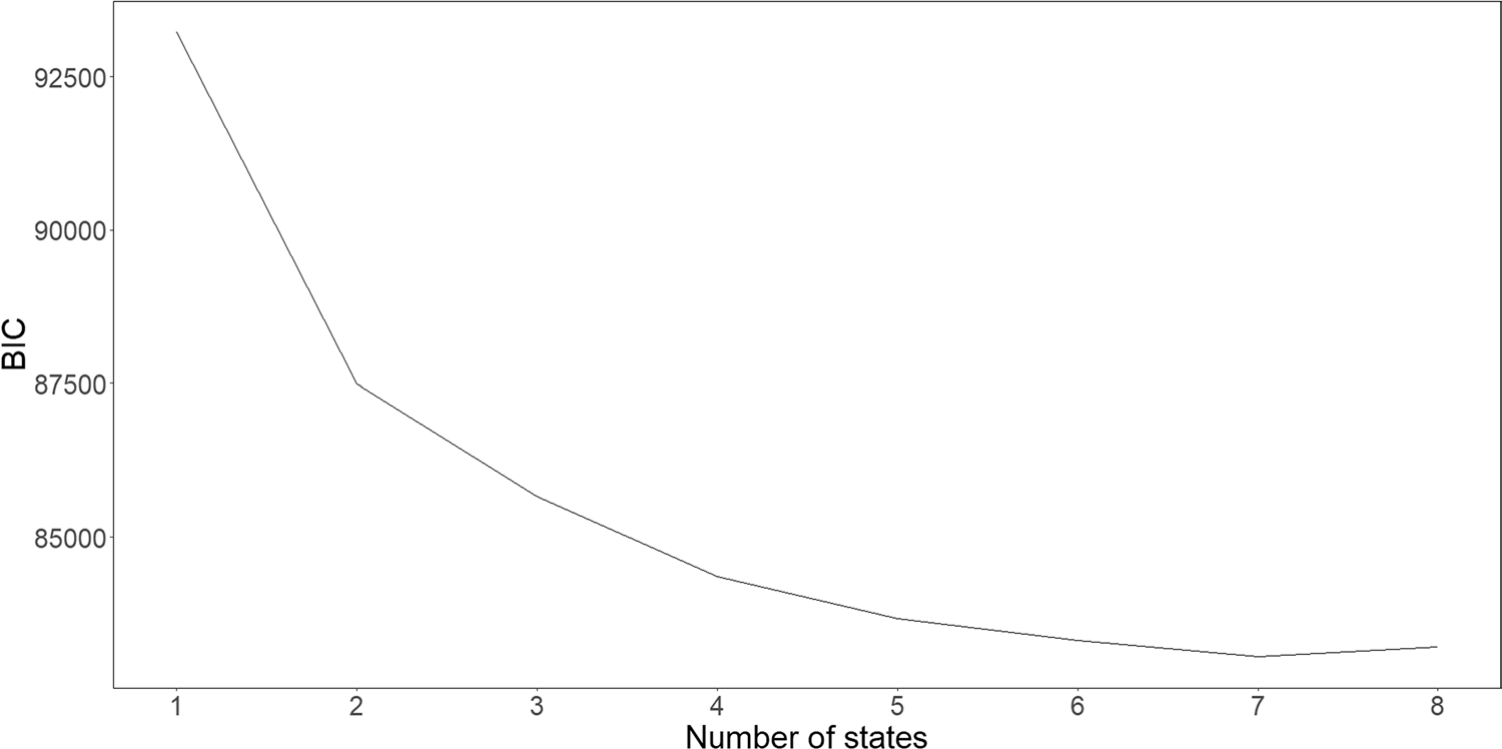
BIC for models with increasing number of states of mental health

Figure 5 shows the BIC for models with an increasing number of categories in the latent variable of mental health. For models with more than three latent states, the AIC and BIC hardly decrease. From seven states onward both criteria increased, indicating a poorer model fit. Thus, we further focused on the two, three and four-state models. We chose the three state model, because models with four or more states contained a very small state (around 1% of the population).

**Fig. 6:**
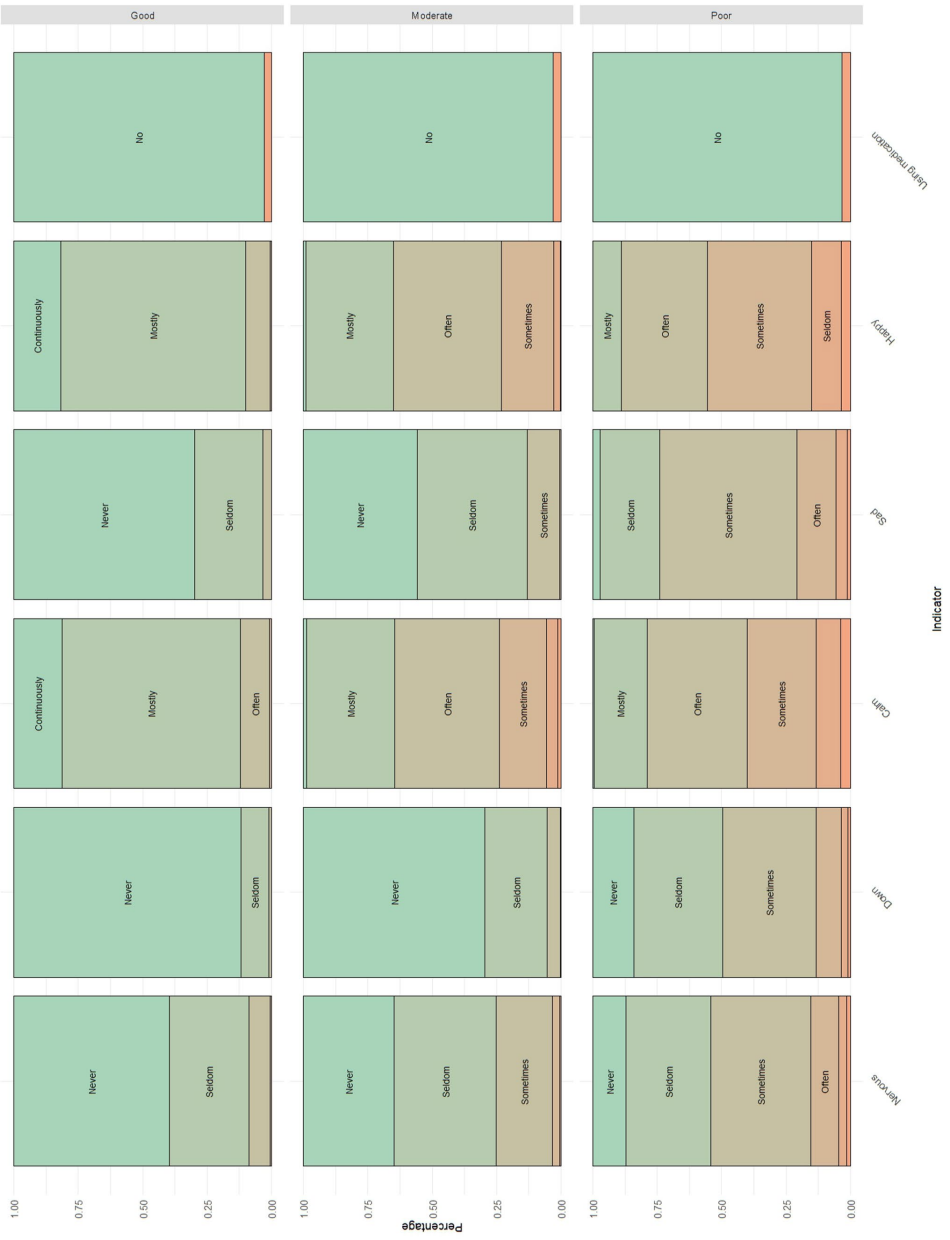
Three mental health states

Figure 6 shows how individuals in the three mental health states were distributed over the categories of the mental health indicators. In the smallest state (26%), the majority of individuals felt negative emotions at least sometimes. Almost half of the respondents in this state reported feeling nervous sometimes or often. In addition, about half of these individuals reported feeling down sometimes or often and about two thirds reported being sad sometimes or often. Positive feelings were less frequent. Just over half of the respondents in this state reported being calm sometimes or most of the times and around 43% reported being happy often or most of the time. Therefore, we labeled this state ‘Poor’ mental health.

Individuals in the largest state (containing around 39% of the study population), reported slightly better mental health that individuals in the ‘Poor’ state. For instance, almost 75% reported being calm sometimes or most of the times and a similar percentage reported being happy sometimes or most of the time. Negative feelings were less frequent as can be seen in Fig. 6. We labeled this state ‘Moderate’.

The remaining respondents (around 35%) were grouped in the state with the highest level of mental health. Approximately 70% of the respondents in this state reported being calm most of the time, and around 19% were continuously calm during the previous two weeks. Similar percentages reported feeling happy most of the time or continuously. Given the relatively high mental health of the individuals in this state, this state was named ‘Good’.

**Fig. 7:**
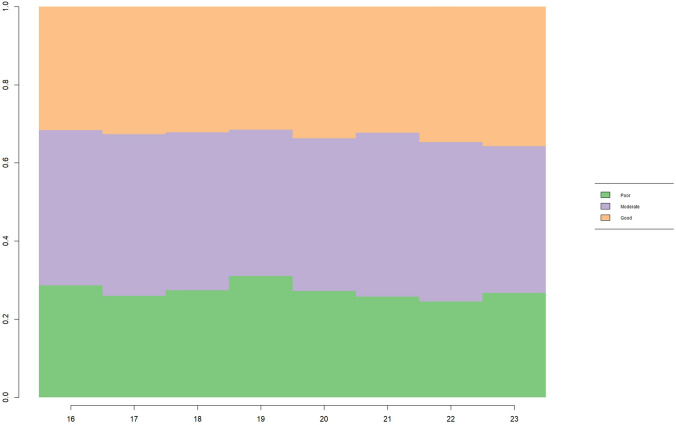
Density plot for mental health states: the share of respondents in each of the states in each year

As for the JDR states, we present density and index plots and transition probabilities for the mental health states. Figure 7 shows the density plot for mental health during the study period. The share of individuals in each of the three mental health states remains relatively stable over time. At the end of the study period, the good mental health group increases slightly while the moderate group decreases slightly.

**Fig. 8:**
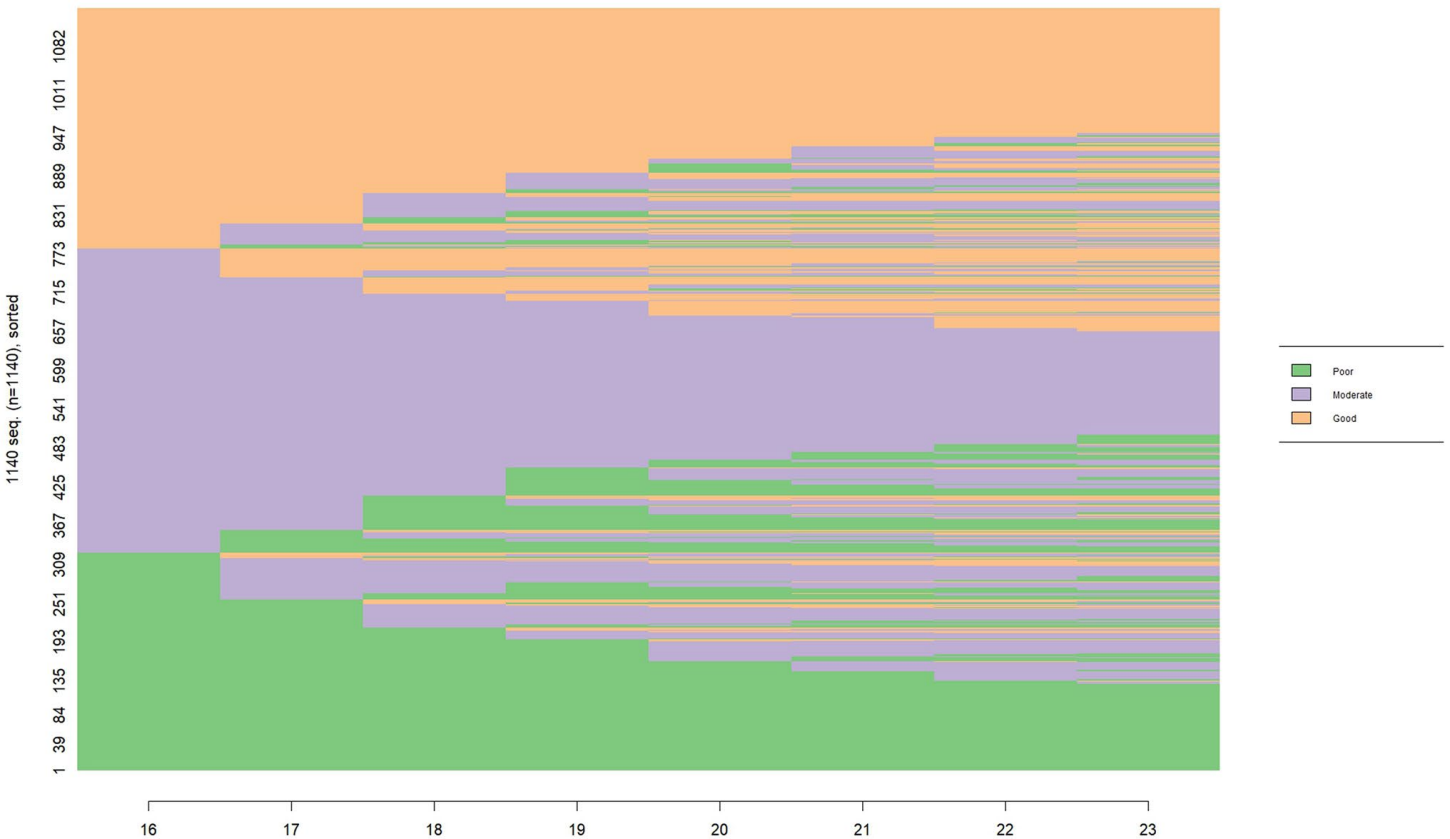
Index plot for the mental health states: each horizontal line represents the transitions of one respondent

Table 2, presenting the transition probabilities, and Fig. 8, representing the index plot, show less stability. The index plot shows a considerable amount of movement between the good and moderate mental health states and the poor and moderate mental health states. Table 2 which shows the average transition probabilities across time points), shows that respondents in the good mental health state are most likely to stay in that state (89.6%). Those who do transition move almost exclusively to the moderate mental health state. Respondents in the poor mental health state are most likely to transition (just 78.7% remains in this state in the next year), and most likely to the moderate state (17.4%). Interestingly, respondents in the moderate mental health state move to the poor mental health state (10.9%) as well as the good mental health state (8.1%).

**Table 2:** Average yearly transitions probabilities between mental health states over the study period

	Health *t*		
Health $t-1$	Poor	Moderate	Good
Poor	78.7%	17.4%	3.9%
Moderate	10.9%	81.0%	8.1%
Good	3.7%	6.7%	89.6%

### The relation between job demands and resources, and mental health

Finally, we investigate the relationship between job demands and resources on the one hand and mental health on the other. Estimates of this relationship are found in the structural part of our HMM model, i.e. the initial state probabilities and the transition probabilities between the six JDR states and the three mental health states discussed above.

#### Initial probabilities

The initial-state probabilities show how JDR and mental health are related at the first measurement, in this case, 2016. Table 3 presents the initial probabilities of being in each of the three mental health states conditional on the JDR state. This table shows that respondents in the ‘Being your own boss’ (40.6%) and especially the ‘Tough job’ (44.7%) states in 2016 were more likely than other JDR states to be in the ’poor’ mental health state in that same year. It also illustrates that respondents in the ‘Dream job’ state were much more likely than respondents in the other states to be in the ‘good’ mental health state (50.4%).

**Table 3:** Initial mental health states for the six JDR states

	Mental health state			
JDR state	Poor	Moderate	Good	Total
Being your own boss	40.6%	32.4%	27.0%	100%
Mentally demanding work on site	25.0%	41.0%	34.0%	100%
Mentally demanding work from home	26.5%	46.8%	26.7%	100%
Dream job	19.3%	30.3%	50.4%	100%
Challenged & appreciated	24.6%	41.3%	34.1%	100%
Tough job	44.7%	32.2%	23.1%	100%
Total	18.4%	44.3%	37.3%	100%

Table 4 shows the initial state probabilities of being in each of the six JDR states conditional on the mental health state. Respondents who were in the poor mental health state in 2016 were more likely to be in the ‘Tough job’ state compared to the entire study population and less likely to be in the ‘Mentally demanding work on site’ state. Respondents who were in the moderate mental health state were somewhat more likely to be in the ‘Challenged & appreciated’ state. Finally, respondents in the good mental health state were more likely to be in the ‘Dream job’, ‘Challenged & appreciated’ and ‘Mentally demanding work on site’ states.

**Table 4:** Initial JDR states for the three mental states

	Mental health state			
JDR state	Poor	Moderate	Good	Total
Being your own boss	10.5%	6.2%	6.3%	7.5%
Mentally demanding work on site	25.0%	30.1%	30.6%	28.8%
Mentally demanding work from home	14.4%	18.6%	13.1%	15.6%
Dream job	5.0%	5.8%	11.9%	7.5%
Challenged & appreciated	21.9%	26.9%	27.3%	25.6%
Tough job	23.3%	12.3%	10.8%	15.0%
Total	100%	100%	100%	100%

#### The influence of mental health on transitions between JDR states

**Fig. 9:**
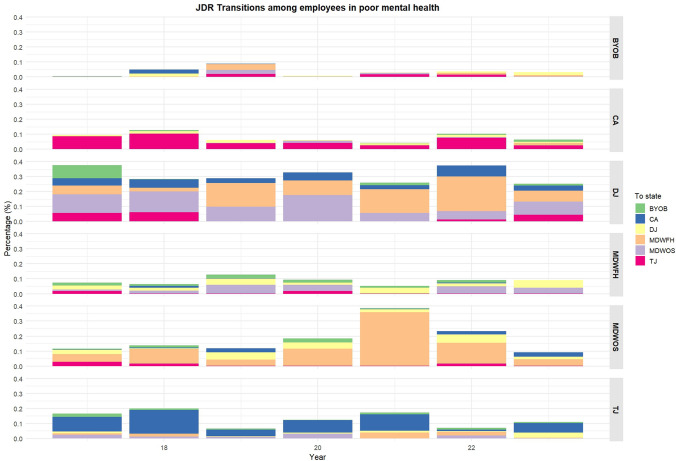
JDR transitions when mental health is poor. The vertical bars in the transition plot represent an individual’s current JDR state. The colored bars demonstrate the transition probability from the current state to other states at each time point when mental health is poor

**Fig. 10:**
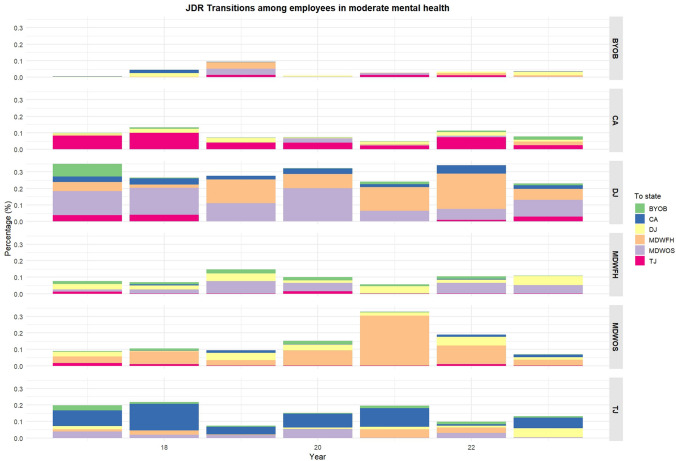
JDR transitions when mental health is moderate.The vertical bars in the transition plot represent an individual’s current JDR state. The colored bars demonstrate the transition probability from the current state to other states at each time point when mental health is moderate

**Fig. 11:**
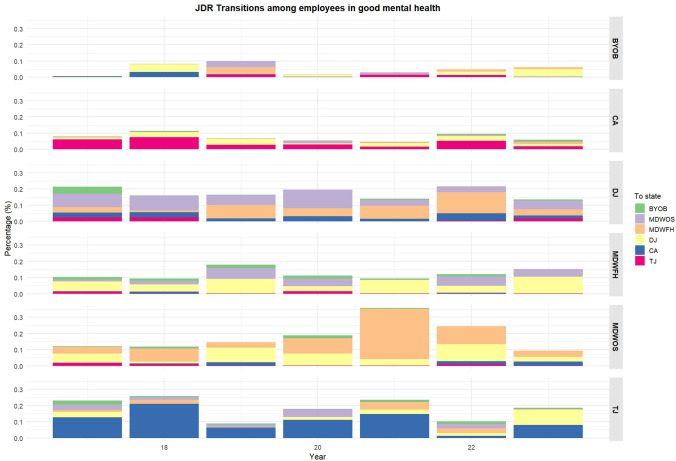
JDR transitions when mental health is good. The vertical bars in the transition plot represent an individual’s current JDR state. The colored bars demonstrate the transition probability from the current state to other states at each time point when mental health is good

To gain more insight into how JDR and mental health affect each other, we discuss the cross-lagged effects, i.e. how the year to year transition probabilities from one JDR state to another vary between different mental health states. Figures 9, 10, 11 illustrate the transition probabilities between the six JDR states for the three levels of mental health: poor, moderate, and good. These figures give us more insight into the over-time change in JDR and the extent to which this change varies according to the level of mental health. The graphs can be read as follows. The label in the gray rectangle represents the JDR state at $t-1$. The top row of each graph thus shows the transitions *from* ‘Being your own boss’ to each of the other five states for each time point (2016 is missing since that is the initial state; 2017 represents the transitions from those who were in ‘Being your own boss’ in 2016 to the other five states in 2017). The height of the bars indicates the total share of respondents who transitioned to another state in a particular year. The colors represent the different states the respondents transitioned to. For example, the blue color in 2018 for ‘Being your own boss’ represents the transition probability from that state in 2017 to ‘Challenged & appreciated’ in 2018 (around 5%).

At first glance, these figures show that the transition probabilities between the six JDR states are quite similar between the three mental health states. However, some differences exist, specifically between poor and good mental health. For instance, we see that transition probabilities for those in the ‘Dream job’ state are high, especially among employees in poor mental health (Fig. 9). These employees are especially likely to transition to one of the mentally demanding states. Especially in 2022 this was the case, likely related to the outbreak of Covid-19 a few years earlier. Further, transition probabilities *from* ‘Tough job’ are higher among those in good health (Fig. 11). They are more likely to transition to ‘Challenged & appreciated’ and in 2023, interestingly, to ‘Dream job’. Lastly, we also see differences between the three mental health groups regarding transitions *from* ‘Mentally demanding work on site’. Employees in good mental health are more likely to transition from that state to ‘Dream job’ than employees in the other two mental health states.

These results suggest that mental health is, to some extent, related to transitions between JDR states. Employees in good mental health are more likely to transition from ‘Tough job’ to ‘Challenged & appreciated’ or even ‘Dream job’ then employees in poor or moderate mental health.

#### The influence of JDR on transitions between mental health states

To illustrate how JDR is related to transitions between mental health states, we focus on the transitions *from* the moderate mental health state *to* the poor or good mental health states. Employees in the moderate state can (and do) move to both the good or poor mental health state, while those in the good or poor mental health state almost exclusively move to the moderate state.^6^ In addition, the moderate mental health state is the largest state at the initial measurement (41.2%).

Table 5 shows the average transition probabilities for respondents who were in moderate health in $t-1$ for the six JDR states. It shows that, conditional on being in the moderate health state at $t-1$, respondents in the ‘Tough job’ state, are most likely to transition to the poor mental health state (14.6% yearly) and least likely to transition to the good mental health state (7.0% yearly). Those in the ‘Mentally demanding work on site’ and the ‘Dream job’ state are least likely to transition to the poor mental health state (9.5% and 9.1% respectively). Respondents in the ‘Dream job’ state are most likely to transition from moderate to good mental health (13.6% yearly).

**Table 5:** Transitions *from* moderate mental health to poor, moderate or good mental health across six JDR states and eight time points

		Mental health *t*		
JDR state $t-1$	Mental health $t-1$	Poor	Moderate	Good
All	Moderate	11.5%	79.4%	9.1%
BYOB	Moderate	12.3%	80.0%	7.7%
CA	Moderate	11.6%	78.7%	9.8%
DJ	Moderate	9.1%	77.4%	13.6%
MDWFH	Moderate	11.9%	79.2%	8.8%
MDWOS	Moderate	9.5%	82.5%	8.0%
TJ	Moderate	14.6%	78.5%	7.0%

**Fig. 12:**
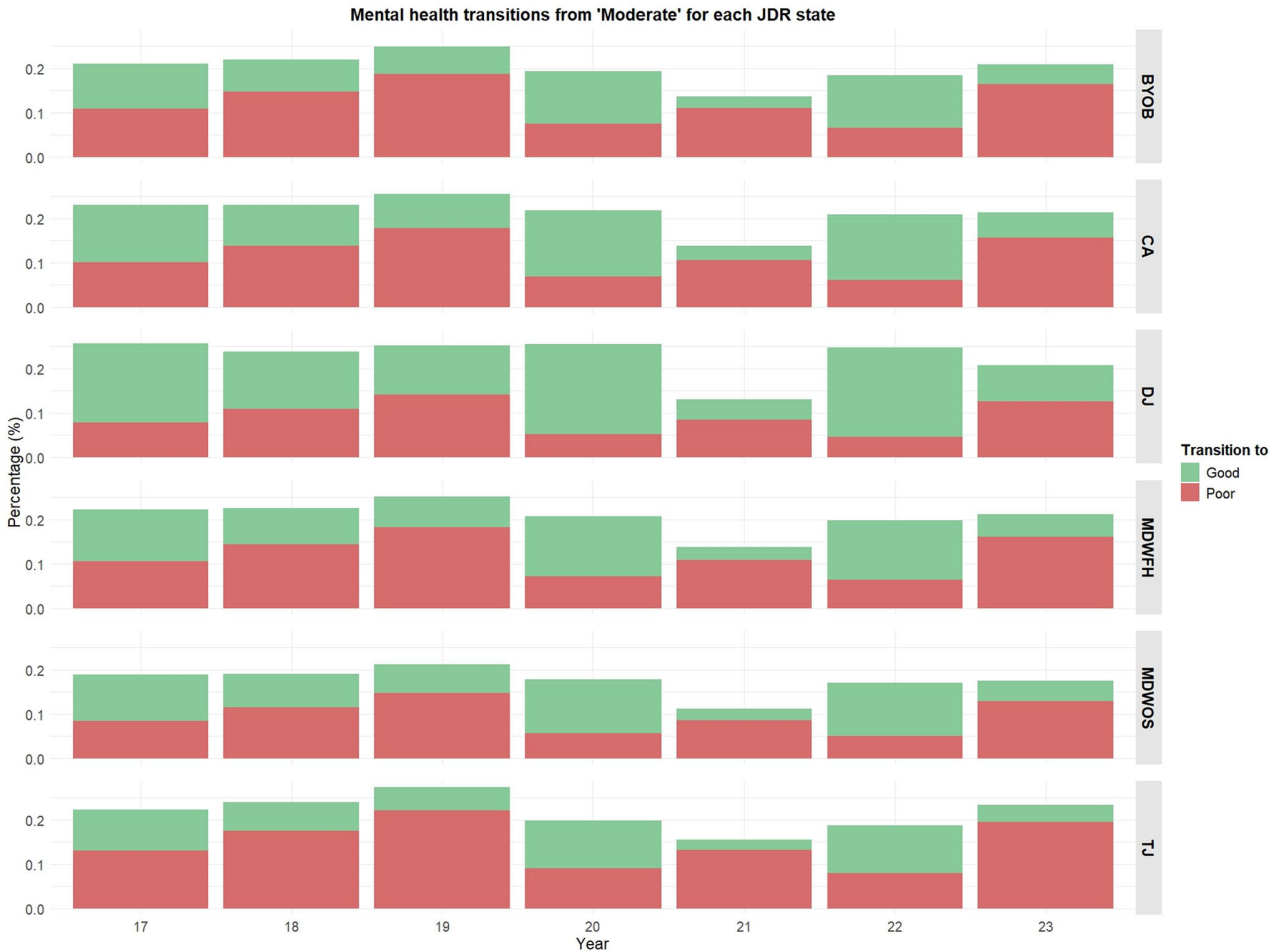
Transition probabilities from moderate mental health to poor or good mental health for the six JDR states

Figure 12 shows the transition probabilities from moderate mental health to poor or good mental health for each of the six JDR states across the years. In general, each year, 15-20% of the respondents who are in the moderate mental health state transition to either the poor or good mental health state. Among those in the ‘Tough job’ state, transitions to the poor mental health state are more common in most years (especially in 2019 and 2021 and 2023). This indicates that since 2017, the mental health consequences of being in the ‘Tough job’ state have deteriorated. It is important to note here that this does not imply that being in this state longer increases one’s likelihood of transitioning to the poor mental health state. It merely suggests that one’s likelihood to transition from moderate mental health to poor mental health increases over the years for individuals being in the ‘Tough job’ state at $t-1$. Transitions from the moderate to the good mental state are especially common among individuals in ‘Dream job’ state. Transitions to poor mental health are much less common (around 5% each year).

## Discussion

In this study, we addressed two research questions. First, which combinations of demands and resources exist among workers in the Netherlands and how often do they occur? And second, how are these combinations of job demands and resources related to mental health? We used a cross-lagged hidden Markov model (HMM). We identified six different combinations of job demands and resources, ranging from ‘Tough job’ with high levels of demands and low levels of resources to ‘Dream job’, with low levels of demands and very high resources. In addition, we found three states of mental health (poor, moderate, good).

Regarding the relationship between JDR and mental health, we found that the extent to which mental health affects transitions between states with different job demands and resources is small. Nevertheless, some notable exceptions do exist. For example, having poor mental health is an obstacle to moving from a ‘Tough job’ to states with fewer job demands and more resources.

In contrast, we found that the improvement or deterioration of mental health is related to job demands and resources experienced by the individual. Among those in moderate mental health, workers in the ‘Tough job’ state were more likely to move to the poor mental health state. In contrast, workers in the ‘Dream job’ state were more likely to move from moderate to good mental health.

Our findings are partly in line with previous research that identified groups of employees that experience different combinations of job demands and resources. For instance, our ‘Tough job’ class, resembles the ‘Demanding’ class in (Van den Broeck et al., 2012). However, there are also differences. For example, we do not find a class that is characterized by low demands *and* low resources, like the ‘Poor’ class in Van den Broeck et al. (2012). Additionally, Portoghese et al. (2025) find similar levels of resources among the five classes that they identify. In our study, we find one state with considerably lower levels of resources than the other classes, namely ‘Tough job’. There are a few possible explanations for these differences. First, the studies of Portoghese et al. (2025) and Van den Broeck et al. (2012) focus on specific occupations, while we do not. Second, the indicators we use are similar but not identical to the indicators used in the other studies. We identified two states with similar demands (mental) but that differed regarding their possibility to work from home. Our results indicate that employees move from the state in which the possibility to work from home is limited to the state in which working from home is more common. Interestingly, this trend started before the COVID pandemic and only slightly accelerated in 2020. We only see slight differences in health transitions between these two states. Transitions from moderate health to poor health are slightly more common among employees that work from home. Interestingly, this is also the case in the pre-COVID years. This suggests that working from home may include a risk for mental health for some employees, perhaps because of a lack of social support.

Our findings are in line with much previous research that has shown that the balance between job demands and resources is related to mental health (Bakker et al., 2023), including recent studies that identified classes of employees with similar job demands and resources and studied differences in well-being between these groups. These studies found that classes defined by high demands, are related to poor well-being, especially when combined with low resources (Portoghese et al., 2025; Van den Broeck et al., 2012), while classes in which high levels of resources are combined with low or even high levels of demands are related to high levels of well-being (Van den Broeck et al., 2012). Similarly, we found that employees in the ‘Tough job’ state were more likely to transition to the ‘Poor health’ state and those in the ‘Dream job’ class more likely to the ‘Good health’ state. However, this study extends previous research both theoretically and empirically. In this research, we assessed whether the association between JDR and mental health is bi-directional: i.e. JDR affect mental health and vice versa, rather than uni-dimensional as assumed in the JDR-model. We assessed whether JDR might affect mental health, and vice versa, therefore considering a healthy worker effect. The results of our study provide some evidence in favor of a healthy worker effect, as the mental health state an individuals is currently in, affects the ways in which individuals move between the current and sequential JDR state. Empirically, we also extend previous research by identifying which combinations of JDR actually occur among employed individuals by using an HMM. This enabled us, for instance, to identify groups of workers with jobs with few job demands and high resources (‘Dream job’) and with jobs with high demands and few resources (‘Tough job’).

The findings of this study are also practically relevant, especially for employers who want to prevent mental health problems among their employees. Our results show that particular combinations of jobs and resources increase the likelihood of decreasing mental health among workers. In particular, workers in the ‘Tough job’ state are more likely to move from moderate mental health to poor mental health. To prevent this, employers should prevent combinations of job demands and resources that represent a tough job. The finding that workers in a similar state, but with lower work pressure and higher resources, often experience transitions from moderate to good health suggests that alleviating work pressure and increasing resources can prevent deterioration of health among these employees. In addition, the finding that individuals in the ‘Mentally demanding work from home’ state are more likely to transition to poor health than individuals in the ‘Mentally demanding work on site’ is practically relevant. Research on the well-being effects of working from home has suggested that it can be beneficial for well-being, for instance because it can increase autonomy, but also detrimental for well-being as it can increase demands, for instance regarding digital up-skilling (Collins et al., 2016). Recent research has distinguished between working from home instead of working on site (‘replacement’) and working from home in addition to working on site (‘extension’) (Yang et al., 2023). This study showed that replacement work from home was related to better mental health while extension working from home was related to worse mental health. Our results, in addition to the results of these studies suggests that working from home has the potential to improve employee well-being, but also to decrease it. Future studies should shed more light on the circumstances that influence the relation of working from home to mental health. The results of such studies could help managers to organize working from home in a way that contributes to employee well-being.

This study demonstrates the advantages of HMMs in the field. We can use these models to estimate profiles (states) of subjects based on a categorical latent variable (person-centered). Simultaneously, HMMs provide transition probabilities between these states over time, looking at $t-1$ relations over time for the categorical latent variable of interest. We also showed how some of the basic assumptions of HMMs can be relaxed, by estimating time-varying transition probabilities. And lastly, we illustrated how a cross-lagged HMM can study the relations between multiple categorical latent variables over time. This can be particularly important in cases of interrelated longitudinal processes, such as health and employment.

Notwithstanding the contributions of this paper, it suffers from a few limitations as well. Firstly, about half of our study population at baseline was lost during follow-up. Since those who dropped-out differed from those who did not regarding, for instance, gender and educational level, this may have influenced our results. For instance, it may have affected the JDR states we found since some combinations of job demands and resources may be more or less common among some demographic groups. Secondly, we aimed to study transitions in JDR and health for a long period of time. Consequently, the COVID-19 pandemic was included in our study period. This may have affected our results in various ways. It is likely that this influenced the JDR states that we identified, in particular the ‘Mentally demanding work from home’ state. It is possible that the COVID-19 pandemic also affected the mental health states that we identified and the relationship between JDR and mental health, although it is less straightforward how this relationship might have been affected. In addition, because of data limitations, we used a general measure of mental health (MHI-5) instead of a work-related measure of mental health, such as burnout. This may have affected our ability to find a relationship between JDR and health. Even though the MHI-5 is a widely used and reliable instrument to measure mental health, it has some drawbacks too. The MHI-5 performs sufficiently well in identifying mood disorders (Rumpf et al., 2001), major depression (Berwick et al., 1991) and some anxiety disorders (Rumpf et al., 2001; Berwick et al., 1991). However the MHI-5 has limited ability to identify several anxiety disorders, substance abuse and somatic symptom disorder (Rumpf et al., 2001). The addition of the variable regarding use of medication to treat anxiety or depression may have resulted in a more reliable estimate of anxiety disorders, including those that the MHI-5 does not sufficiently identify. Despite this, several kinds of employment-related mental health disorders might have been missed. Consequently, the results of our study may be an underestimation of the ways in which job demands and resources are associated with mental health.

In short, this study illustrates how HMMs can be used to study two interrelated longitudinal processes and advances our understanding of how JDR and mental health affect each other theoretically and empirically. We find that, in line with earlier research, JDR affects mental health. People that perform jobs in which demands cluster and with few resources more often experience deteriorating mental health. Conversely, people that perform jobs in which demands are relatively low and many resources cluster more often experience improving health. We also found some evidence that health affects JDR. For instance, having poor mental health seems to prevent moving from a ‘Tough job’ to states with fewer job demands and more resources.

## Footnotes

1 https://www.vzinfo.nl/overspannenheid-en-burn-out/internationaal.

2 https://www.vzinfo.nl/overspannenheid-en-burn-out/leeftijd-en-geslacht.

3 This means that for each person, the likelihood of belonging to each of the identified categories is estimated.

4 With people we mean person-years. The percentages refer to a share of the total amount of observed person-years, since every person is in the data more multiple (at least four) years.

5 The AIC values for the AIC were very similar and are therefore not shown.

6 In the online Appendix the transition probabilities between the six JDR states are shown for all three mental health states.
